# A Component-Level Defect Detection and Real-Time Localisation Method for Photovoltaic Arrays Using UAV-Based Infrared Imagery

**DOI:** 10.3390/s26123736

**Published:** 2026-06-11

**Authors:** Hui Peng, Yongqiang Cui, Di Bai, Qian Huang, Xiaoli Chen

**Affiliations:** 1 College of Electronics and Communication Engineering, Kashi University, Kashi 844008, China; peng_9966@163.com; 2 College of Electronic and Information Engineering, South-Central Minzu University, Wuhan 430079, China; cuiyq@mail.scuec.edu.cn (Y.C.); dibai@scuec.edu.cn (D.B.); 2022120316@mail.scuec.edu.cn (Q.H.); 3 School of Electronic Information, Wuhan University, Wuhan 430072, China

**Keywords:** photovoltaic defect detection, YOLOv8-OBB, infrared image, UAV inspection, component-level localisation, Hough transform, K-means clustering

## Abstract

Defects in photovoltaic (PV) modules, including hotspots, shading, and diode failures, significantly reduce power-generation efficiency and pose safety risks. This study proposes a real-time detection and localisation framework for PV defects based on infrared images acquired by unmanned aerial vehicles (UAVs). A dedicated dataset of 5583 infrared/visible images was constructed under standardised acquisition conditions. An improved rotating-bounding-box detector, termed YOLO-CLO, was developed upon YOLOv8-OBB by introducing a lightweight C3m module and a shared-convolution LSCD-OBB detection head. The proposed detector attains 99.1% mAP@0.5, 96.7% mAP@0.5:0.95, and 59.88 FPS with only 8.52 M parameters and 23.6 GFLOPs, outperforming the baseline in both accuracy and efficiency. A multi-feature image-processing pipeline combining gradient, grayscale, temperature, and morphological cues identifies hotspots, diode failures, and obstructions with detection accuracies of 96.97%, 100%, and 88.89%, respectively. A component-level localisation strategy integrating GNSS metadata, the Hough transform, and an improved K-means clustering algorithm accurately recovers the row–column index of each defective module within an array. Comparative experiments against YOLOv5 and Faster R-CNN confirm the superiority of the proposed framework. The method offers low hardware dependency and is suitable for engineering deployment in large-scale PV power stations.

## 1. Introduction

Growing concerns over energy security and the accelerating global transition to clean energy have positioned photovoltaic (PV) power generation as a cornerstone of the low-carbon energy system. According to the 2024 BP World Energy Outlook [1] and the World Energy Statistics Yearbook 2024 [2], newly installed solar and wind capacity reached 276 GW globally in 2023, of which 75% (346 GW) came from solar. Cumulative PV capacity reached 1412.09 GW, corresponding to a year-on-year increase of 32.4%.

PV panels operate outdoors for extended periods and are therefore susceptible to environmental stressors, which generate typical defects such as hotspots, partial shading, and bypass-diode failures [3,4]. These defects reduce generation efficiency and may cause thermal runaway or fire. Efficient and low-cost defect-detection technology is thus critical for maintaining plant performance over its lifetime. Existing approaches are broadly divided into two categories: (i) monitoring based on electrical characteristics (output voltage, current, and power) [5,6,7] and (ii) detection based on infrared image analysis. Electrical methods require thousands of sensors for large plants, leading to prohibitive cost and declining efficiency at scale. Image-based methods, in contrast, are non-contact, deployment-friendly, and have become the mainstream research direction.

Traditional computer-vision approaches have been widely investigated. Tsanakas et al. [8] applied Canny edge detection on thermal images for hotspot identification. Ngo et al. [9] combined K-means clustering with DBSCAN for contour extraction, but edge information is easily obscured in cluttered backgrounds. Chen et al. [10] employed single-channel thresholding and colour-space statistics, which improved generalisation yet still suffered from information loss due to statistical descriptors. With the rise in deep learning, Wu et al. [11] adopted an improved LeNet-5 for fault classification, and Greco et al. [12] applied the YOLO framework across 18 datasets. Deep models outperform traditional pipelines in feature representation but typically require millions of parameters [13], imposing significant hardware costs. Existing methods therefore face a dual bottleneck: traditional algorithms lack accuracy under complex backgrounds, whereas deep networks are too heavy for edge deployment, and few studies achieve component-level localisation beyond coarse bounding-box detection. More specifically, traditional pipelines rely on hand-crafted thresholds whose stability degrades when irradiance, viewing angle, or array layout change between sites, while deep detectors that achieve state-of-the-art accuracy on benchmark PV datasets (e.g., YOLO-based and Faster R-CNN-based variants) generally adopt horizontal bounding boxes that include background pixels of neighbouring arrays, raising both false-detection and miss rates in densely arranged plants. Moreover, the majority of published works report only image-level or array-level outputs and do not return the row–column index of the faulty module that field crews actually need for on-site replacement, which leaves a concrete gap between reported accuracy and operational utility.

To address these gaps, this study proposes a two-stage automatic defect-recognition method that combines a lightweight deep detector with traditional image-processing cues, followed by a component-level localisation strategy. The main contributions are summarised as follows:

(1) A UAV-based infrared inspection platform is established and a large-scale PV infrared image dataset is constructed under standardised conditions and released for non-commercial research use.

(2) An improved rotating-box detector YOLO-CLO is developed upon YOLOv8-OBB, in which a lightweight C3m module replaces the original C2f, and a shared-convolution LSCD-OBB detection head is introduced. The detector enables real-time, high-precision extraction of individual PV arrays and is complemented by a multi-feature thresholding pipeline for defect classification.

(3) A component-level row–column localisation method is proposed, integrating UAV GNSS metadata, the Hough transform, and an improved K-means clustering scheme, enabling pixel-to-module mapping within any detected faulty array. Compared with prior YOLO-based PV detectors and Hough-based line-extraction methods, the novelty of this work lies in three specific design choices rather than in the use of these building blocks themselves: (i) the C3m module re-engineers the C2f/C3 design by replacing one CBS sub-block with a plain convolution and inserting a Mish activation after branch fusion, giving a different parameter–accuracy operating point from existing C2f variants; (ii) the LSCD-OBB head shares the two 3 × 3 Conv_GN blocks across the regression and classification branches while keeping the angle branch independent and introduces a learnable per-head scale factor to compensate for the resulting feature-scale mismatch—a configuration not adopted in standard YOLOv8-OBB; and (iii) the localisation module couples UAV-logged GNSS metadata, Hough-line extraction and prior-knowledge-initialised K-means clustering into a unified pipeline that returns absolute array coordinates and intra-array row–column indices, addressing the operational gap identified above.

## 2. Materials and Methods

### 2.1. UAV Inspection System and Dataset Construction

**Figure 1:**
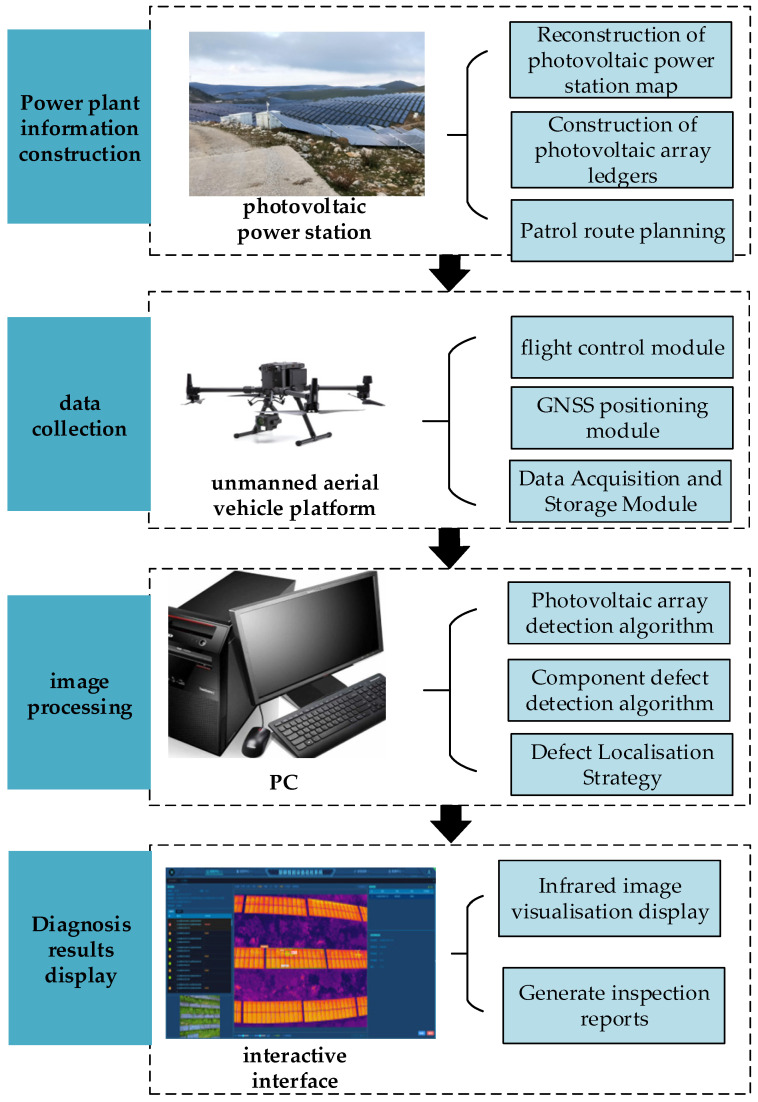
Architecture of the UAV-based PV inspection system.

UAV-based aerial thermography is adopted as the primary inspection modality, significantly reducing the manpower and field time required by conventional manual or electrical inspection. UAVs equipped with infrared cameras capture thermal images of PV arrays, which are transmitted to a ground processing unit executing the detection and localisation algorithms. The resulting diagnosis is relayed back to the plant for maintenance scheduling. The overall system architecture is illustrated in Figure 1.

Two UAV platforms were deployed: a DJI M300 (DJI, Shenzhen, China) equipped with a Zenmuse H20T dual-spectrum camera (DJI, Shenzhen, China) and an Autel EVO II Dual 640T (Autel Robotics, Shenzhen, China). Both platforms captured infrared images at 640 × 512 pixels and simultaneously logged geographic coordinates (latitude, longitude, altitude) at acquisition, providing the metadata required for subsequent physical localisation. Pre-planned flight paths were designed according to plant layout to ensure full coverage without omission. Data were collected under clear-sky, fog-free conditions; flight altitude was maintained between 6 m and 20 m; and the infrared camera axis was held perpendicular to the module plane to minimise perspective distortion. For reproducibility, the intrinsic parameters of the two infrared cameras (focal length, sensor pitch, field of view) and the thermal sensitivity (noise-equivalent temperature difference, NETD) follow the manufacturer technical specifications of the DJI Zenmuse H20T and the Autel EVO II Dual 640T, respectively, and are referenced from the official platform datasheets. Before each mission the in-camera non-uniformity correction (NUC) routine was executed to ensure radiometric consistency, and the camera was warmed up for several minutes after power-on to reduce drift in the reported temperatures.

**Figure 2:**
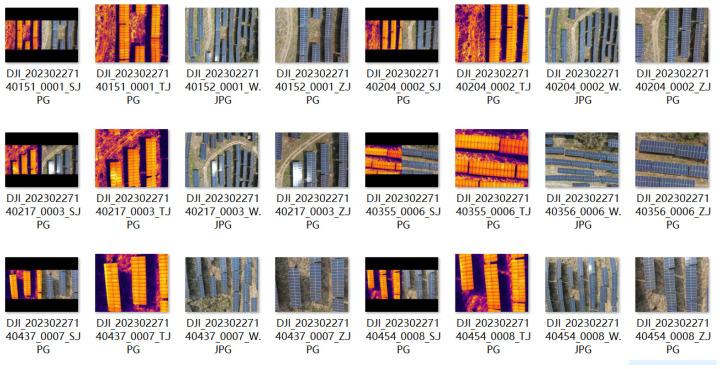
Representative samples from the constructed PV infrared dataset.

Under this standardised protocol, 5583 paired visible-light and infrared images were collected (Figure 2). The dataset offers three strengths: (i) homogeneous weather conditions ensuring strong data comparability; (ii) standardised acquisition parameters ensuring geometric consistency; and (iii) comprehensive spatial coverage ensuring sample representativeness, making it well-suited for PV condition monitoring and defect analysis. Oriented bounding-box annotations of complete PV arrays were performed manually using the rolabelImg tool by two trained annotators, and a third independent reviewer cross-checked every label to enforce annotation-quality control. The dataset is split at the image level into training, validation and test sets in a fixed 8:1:1 ratio; to mitigate the risk of optimistic accuracy estimates caused by acquisition similarity, images captured along the same UAV flight path or from highly overlapping viewpoints of the same array were assigned to the same subset whenever possible, so that closely matched scenes do not appear simultaneously in training and test. Defect-level annotation is not required by the proposed multi-feature pipeline, since defects are identified by physics-based thresholds on the cropped arrays rather than by a learned classifier; the small set of defects used for the per-class evaluation in Table 1 was annotated by visual inspection of the test-set infrared images. For the plant-ledger interaction shown in Figure 1, the ledger is a pre-stored table of the geographic centroids of all PV arrays in the plant; when a faulty array is detected, the localisation module queries this ledger with the GNSS-derived position to retrieve the corresponding array identifier, while the module-level row–column index is computed locally from the cropped array image and does not require any prior ledger entry.

### 2.2. Defect Categories and Thermal Signatures

**Figure 3:**
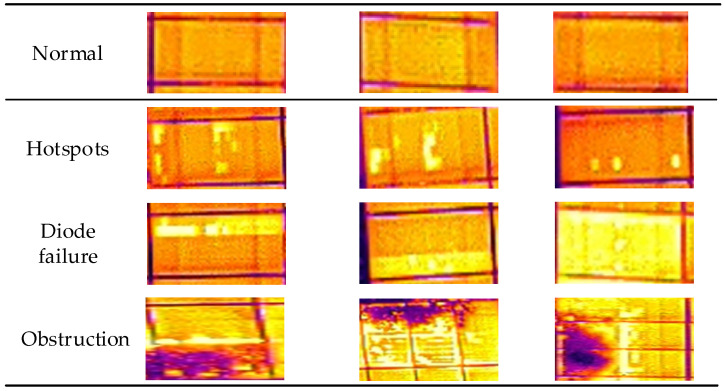
Thermal signatures of PV components with different defect types.

Three of the most common and safety-critical PV defects are addressed: (1) Hotspots appear as localised bright regions with regular circular or elliptical shape, distinctly higher grayscale values than surrounding normal areas, steep thermal gradients, and sharply defined boundaries. (2) Diode failures manifest as narrow or elongated high-temperature regions at solder joints or busbars, with bright cores and gradually decreasing peripheral gradients; heat diffusion may slightly blur the boundary, yet grayscale contrast remains evident. (3) Obstructions appear as large, low-intensity regions whose shape mirrors the occluding object, with grayscale values significantly lower than surrounding unobstructed areas and distinct gradient edges. A comparison between defective and normal components is shown in Figure 3.

## 3. Real-Time Rotating PV Array Detection

### 3.1. Overall Framework

**Figure 4:**
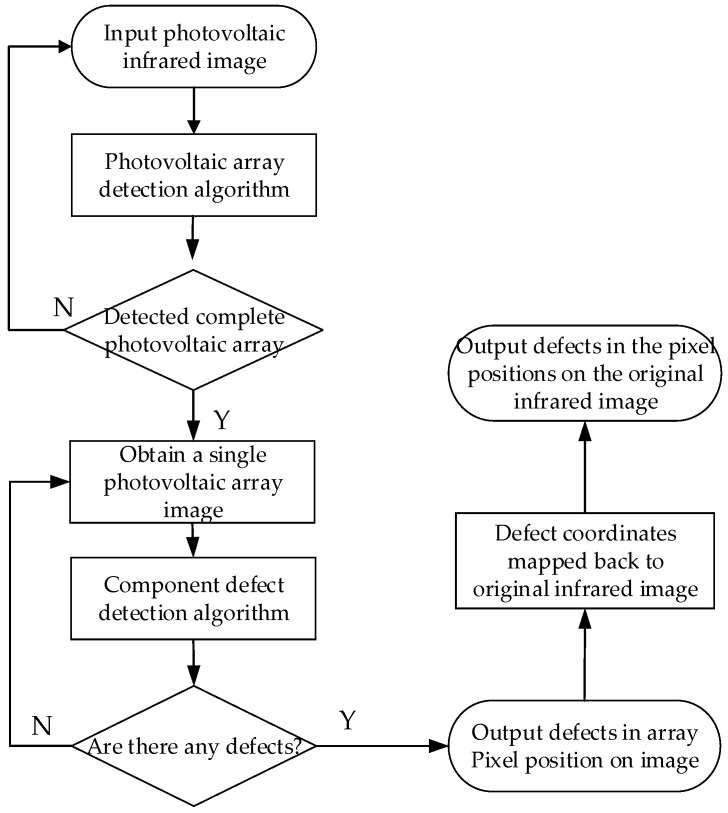
Flowchart of the proposed detection and localisation framework.

The algorithmic pipeline is shown in Figure 4. Upon receipt of a UAV infrared image, the improved YOLO-based rotating detector locates all PV arrays and outputs their oriented pixel coordinates. Because a single image typically contains multiple arrays, each detected region is cropped through an affine transformation for independent analysis. The cropped patches are then processed by the multi-feature defect-detection pipeline. Arrays free of defects are skipped; otherwise, defect pixel coordinates are recorded and mapped back to the original image via the inverse affine transformation for global visualisation. The process repeats until all arrays in the image and all images in the mission have been processed.

### 3.2. PV Array Extraction

#### 3.2.1. Rotating Detector Design

Raw UAV infrared images often contain multiple complete or partial PV arrays together with substantial background. Detecting defects directly on such images is vulnerable to background noise, especially when horizontal bounding boxes (HBB) [14] are used, as they introduce extraneous regions that degrade accuracy. Oriented bounding boxes (OBB) [15] fit rotated targets more tightly and avoid overlap among densely arranged arrays and are therefore adopted in this study.

**Figure 5:**
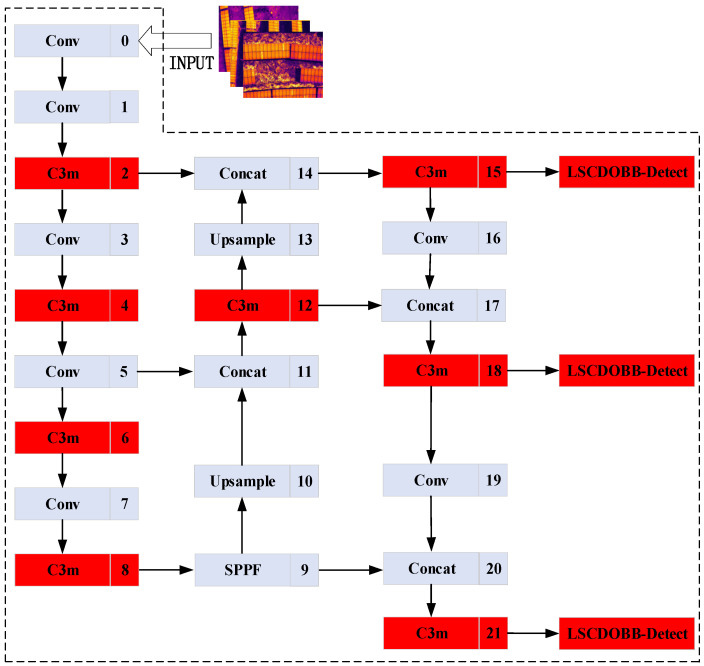
Network architecture of the proposed YOLO-CLO detector.

YOLOv8-OBB is the first YOLO release to natively support rotating-object detection and demonstrates strong robustness. Upon this baseline, two improvements are integrated: a lightweight C3m module replacing C2f, and a shared-convolution LSCD-OBB detection head. The resulting model, YOLO-CLO, reduces computational cost while improving accuracy (Figure 5). YOLOv8-OBB was selected over alternative oriented detectors for three practical reasons. First, two-stage rotated detectors such as Oriented R-CNN and ReDet typically deliver higher accuracy on large benchmarks at the cost of substantially more parameters and lower inference speed, which is unfavourable for UAV-side or edge deployment. Second, anchor-free rotated detectors with separate angle-classification heads (e.g., R3Det, S2A-Net) are competitive on aerial imagery but rely on more complex angle-encoding schemes that complicate lightweighting of the head; YOLOv8-OBB integrates angle prediction natively as a regression branch, which is well-matched to the shared-convolution LSCD-OBB design proposed here. Third, YOLOv8-OBB has a mature, open-source training and deployment toolchain and represents the strongest single-stage rotating-box baseline at the time of this study, providing a fair and reproducible reference for the proposed improvements.

#### 3.2.2. Lightweight C3m Module

YOLOv8 extensively adopts the C2f module, which doubles the input channel dimension via convolution, passes the feature map through multiple bottleneck layers, concatenates intermediate outputs with the original feature map, and compresses channels to the target count through a final convolution. Compared with the earlier C3 module, C2f enriches gradient flow via additional cross-layer connections, enhancing feature fusion at the cost of increased complexity.

**Figure 6:**
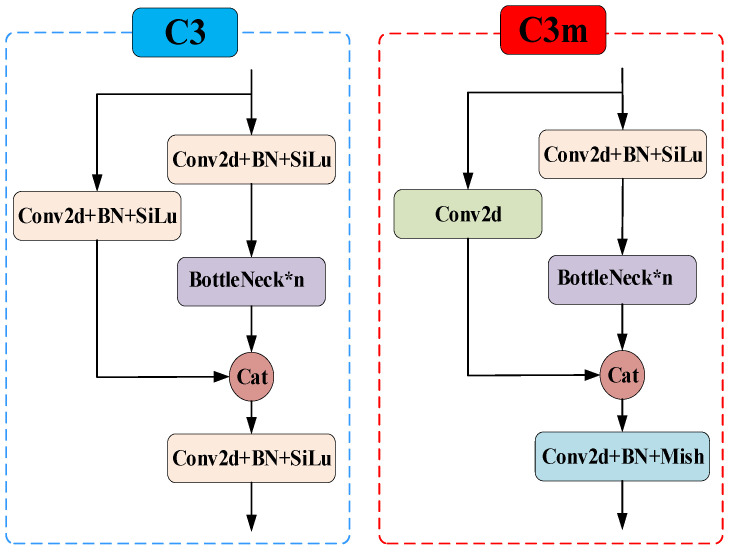
Structural comparison between the C3m and C3 modules.

To balance accuracy against efficiency, this study proposes the C3m module, which combines the merits of C3 and C2f. C3m contains two branches: one branch applies a single 2D convolution, while the other passes through a CBS block (Conv2d + BN + SiLU) followed by n bottleneck layers, with n controlled by the network depth/width multipliers. The branch outputs are concatenated and fused by a convolutional layer with Mish activation, compressing the channel count to half of the original. As shown in Figure 6, C3m replaces one CBS block with a plain Conv2d to lower complexity, while the Mish activation following branch fusion smooths information flow and reduces feature redundancy, enabling effective features to propagate deeper. The computational advantage of C3m can be made more rigorous as follows. For an input feature map with C channels, the C2f module uses two CBS blocks (one for branch splitting and one for the final fusion), giving an approximate convolutional cost of 2C^2^ from these two 1 × 1 projections plus n bottleneck operations. C3m replaces the first CBS with a plain Conv2d that omits the BN and activation overhead and halves the output channels of one of the branches, so the dominant fusion cost is reduced from 2C^2^ to roughly 1.5C^2^; with the same n bottleneck layers, the per-module FLOPs decrease accordingly. This is consistent with the ablation in Table 2, where replacing C2f with C3m at every stage of the backbone reduces overall parameters by 8.43% and GFLOPs by 8.16%, while mAP@0.5:0.95 increases by 0.2 pp—indicating that the saved capacity is not informative for the PV array task and that the smoother Mish activation does not harm representation quality.

#### 3.2.3. Lightweight Shared-Convolution Rotating Detection Head (LSCD-OBB)

Rotating boxes require an additional angle-prediction branch on top of the conventional regression and classification branches. The baseline YOLOv8-OBB uses three detection heads at different scales, each containing three independent branches: the bounding-box regression branch locates the object, the classification branch predicts category and confidence, and the angle branch estimates the rotation relative to the horizontal axis. Although functionally collaborative, these branches replicate convolutional computations and inflate the parameter count.

Three targeted improvements are therefore introduced. First, the Conv module (Conv2d + Normalisation + SiLU) is refined by replacing batch normalisation (BN) with group normalisation (GN) [16]. GN divides channels into groups and computes statistics within each group, decoupling normalisation from batch size and thus suiting hardware-constrained settings. For an input feature map $x\in R^{N\times C\times B\times W}$, the normalisation is formulated as:

(1) $${\hat{x}}_{i}=\frac{1}{\sigma_{i}}(x_{i}-\mu_{i})$$

(2) $$\mu_{i}=\frac{1}{m}\sum_{k\in S_{i}}x_{k}$$

(3) $$\sigma_{i}=\sqrt{\frac{1}{m}\sum_{k\in S_{i}}{\left(x_{k}-\mu_{i}\right)}^{2}+\varepsilon }$$

where ε is a small constant, $S_{i}$ is the set of pixels used for statistics, and m is its cardinality. BN and GN differ only in the definition of $S_{i}$: for BN, $S_{i}$ contains all pixels sharing the same channel index; for GN, $S_{i}$ contains all pixels within the same channel group (group size C/G).

**Figure 7:**
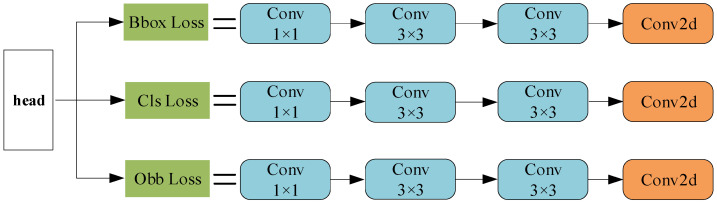
Structure of the proposed LSCD-OBB detection head with Conv_GN blocks and shared convolutions between the regression and classification branches.

Second, since each of the three branches contains two identical 3 × 3 Conv_GN blocks, parameters are shared between the regression and classification branches, while the angle branch remains independent due to its higher sensitivity to spatial cues. Third, to reconcile the feature-scale mismatch that emerges when convolutions are shared across heads, a learnable scale factor is introduced on the regression branch. Initialised to 1.0, the scale factor is updated via backpropagation during training, enabling each head to adaptively calibrate its regression range. As the classification and angle branches are relatively insensitive to feature-map scale, no scale factor is applied to them. The revised detection head is shown in Figure 7. Conceptually, sharing the two 3 × 3 Conv_GN blocks between the regression and classification branches constrains them to learn a common intermediate feature representation, which in principle reduces the representation capacity available to each task individually. In the PV array detection setting, however, the regression and classification objectives are tightly correlated—the same oriented PV-panel signature determines both the bounding box and the (single foreground) class—so a shared feature is well-aligned with the task structure rather than detrimental to it. The learnable per-head scale factor on the regression branch further compensates for any residual scale mismatch caused by sharing. The angle branch is deliberately kept independent because rotation prediction is more sensitive to fine-grained spatial cues, where a shared representation could conflict with the rotation-invariant features preferred by classification. The ablation in Table 2 confirms that the LSCD-OBB head reduces parameters by 16.87% relative to YOLOv8-OBB without degrading mAP@0.5 or mAP@0.5:0.95, indicating that the sharing scheme preserves the effective representation capacity for this task.

#### 3.2.4. Experiments and Ablation Study

All experiments were conducted on a Linux workstation with PyTorch 1.8.1, CUDA 11.4, Python 3.8.15, an Intel Xeon E5-2650 v4 CPU, and an NVIDIA GeForce GTX 1080 Ti GPU. Hyperparameters are listed in Table 1. From the 5583 collected images, 1579 containing one or more complete arrays were manually selected and augmented to form a dataset of 3158 images, split 8:1:1 into training (2526), validation (316) and testing (316) sets, with a DJI:Autel ratio of approximately 6:4 in every subset.

Performance is evaluated using the number of parameters, GFLOPs, frames per second (FPS), and mean average precision (mAP). Parameters reflect spatial complexity, while GFLOPs quantify the floating-point operations per forward pass; smaller values of both favour edge deployment. FPS measures real-time capability. mAP@0.5 is computed at an IoU threshold of 0.5, whereas mAP@0.5:0.95 averages AP over IoU thresholds from 0.5 to 0.95 at 0.05 intervals. Precision, recall, AP, and mAP are defined as:

(4) $$P=\frac{TP}{TP+FP}\times 100\%$$

(5) $$R=\frac{TP}{TP+FN}\times 100\%$$

(6) $$A_{AP}=\int_{0}^{1}P(r)\;dr$$

(7) $$mAP=\frac{\sum_{i=1}^{n}AP_{i}}{n}$$

Here TP, FP, and FN denote correct, false, and missed predictions, respectively, and K is the number of object categories. Ablation results are reported in Table 3.

**Figure 8:**
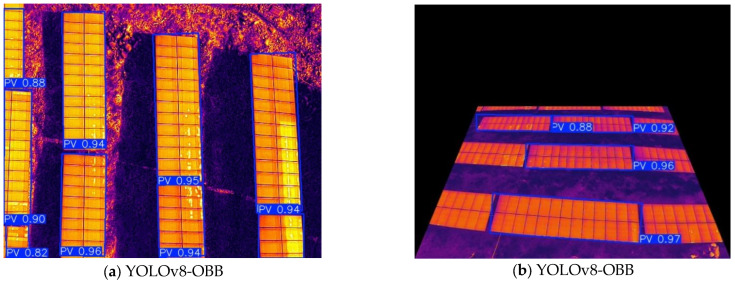

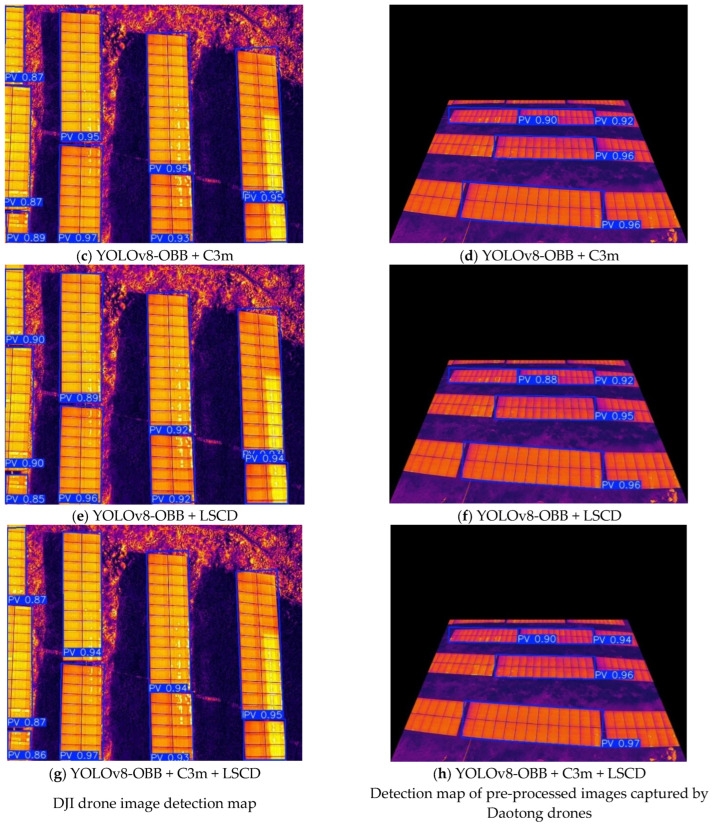
Detection visualisations of different models on DJI and Autel UAV imagery.

Replacing C2f with C3m preserves mAP@0.5 (99.0%) and raises mAP@0.5:0.95 by 0.2 pp, while reducing parameters and GFLOPs by 8.43% and 8.16%, respectively, and boosting FPS by 11.37%. Integrating the LSCD-OBB head alone improves mAP@0.5 to 99.1% and mAP@0.5:0.95 to 96.7%, with a 16.87% parameter reduction and a 9.51% speed gain. The full YOLO-CLO model combines both enhancements, reaching 99.1% mAP@0.5 and 96.7% mAP@0.5:0.95 with only 8.52 M parameters, 23.6 GFLOPs, and 59.88 FPS. Against the baseline, this corresponds to a 25.31% parameter reduction, a 19.73% drop in GFLOPs, and a 17.37% speed increase. Detection visualisations are shown in Figure 8. Although the absolute mAP@0.5 improvement over the baseline is modest (0.1 pp), the practical significance of the YOLO-CLO design should be assessed jointly with its efficiency: at the same or higher detection accuracy, the parameter count and FLOPs are reduced by approximately one quarter and one fifth respectively, and the inference throughput is increased by 17.37%. For UAV-side or edge deployment, where memory footprint and per-frame latency dominate the deployment envelope rather than the last few tenths of a mAP point, this trade-off translates directly into longer inspection missions per battery, lower-cost on-board hardware, and tighter coupling between acquisition and detection. The FPS values reported here are measured for end-to-end neural-network inference of the YOLO-CLO detector on the test workstation (NVIDIA GTX 1080 Ti, batch size 1, input 640 × 640, including pre- and post-processing of the detector). The full detection-plus-localisation pipeline, which additionally performs affine cropping, the multi-feature thresholding pipeline, Hough-line extraction, K-means clustering, GNSS matching and report writing, runs at a lower throughput in real deployment; its end-to-end timing is discussed in the section on limitations.

### 3.3. Multi-Feature Defect-Detection Pipeline

#### 3.3.1. Algorithm Design

**Figure 9:**
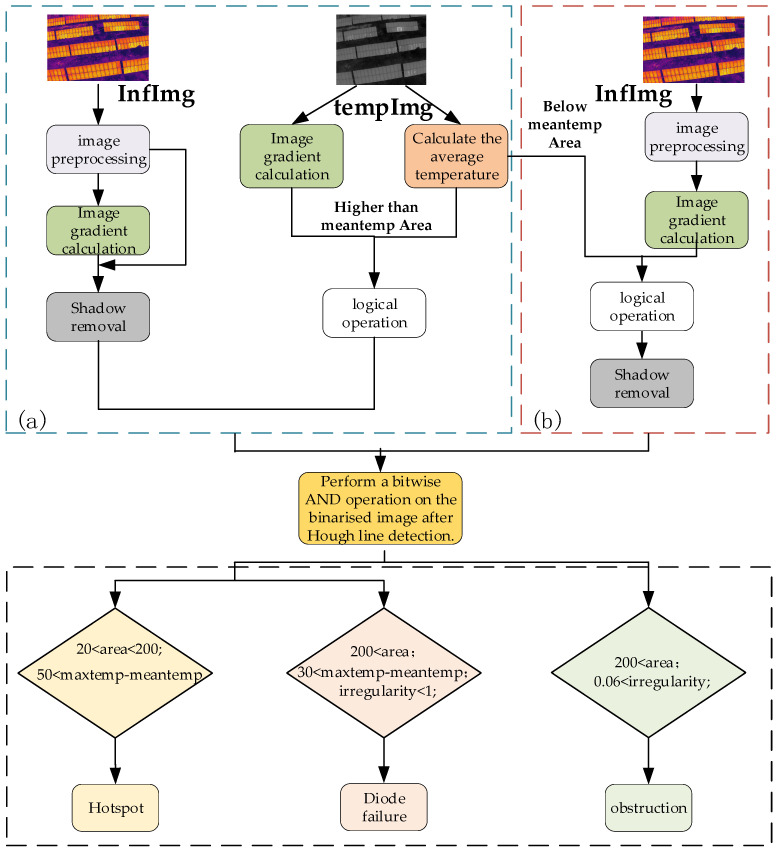
Workflow of the multi-feature defect-detection algorithm: (**a**) the above-mean-temperature branch, which extracts regions hotter than the array mean for hotspot and diode-failure detection; (**b**) the below-mean-temperature branch, which extracts regions cooler than the array mean for obstruction detection.

Guided by the distinct thermal and textural signatures of the three defect types, a five-stage defect-detection pipeline is designed (Figure 9): image pre-processing, gradient-threshold segmentation, grayscale-based shadow removal, average-temperature thresholding, and defect classification.

(1) Image Pre-processing. Normal PV regions exhibit gradual grayscale transitions corresponding to low-frequency content, whereas edges, noise, and defects correspond to high-frequency content. As some minor defects present weak contrast, the original grayscale image is enhanced jointly in the general and high-frequency domains, and the two enhanced versions are fused through a weighted sum (weight = 0.5) to improve both detail visibility and overall contrast. Contrast-Limited Adaptive Histogram Equalisation (CLAHE) [17] is adopted, operating through histogram clipping and interpolation. For a sub-block of size M × N, the local mapping is defined by:

(8) m(i)=(L−1)⋅CDF(i)   i=0,1,2,…,L−1

where *CDF*(*i*) is the cumulative distribution of the sub-block grey-level histogram and *L* is the number of grey levels. The derivative of *CDF*(*i*) is the normalised histogram *H*(*i*), whose slope *S* is bounded to limit contrast:

(9) $$H_{max}=\frac{S_{max}}{L-1}$$

Histogram content exceeding H_max is not discarded but redistributed uniformly across the grayscale range to preserve the total histogram area. Interpolation (corner, boundary, and bilinear) mitigates block artefacts across sub-blocks.

(2) Gradient-Threshold Segmentation. Defect edges commonly exhibit high gradient magnitudes. The Sobel operator [18] is chosen for its low cost and noise robustness:

(10) $$G(x,y)=\sqrt{G_{x}^{2}(x,y)+G_{y}^{2}(x,y)}$$

(11) $$\theta (x,y)=\arctan\left(\frac{G_{y}(x,y)}{G_{x}(x,y)}\right)$$

Otsu’s method [19] is then applied to automatically select the binarisation threshold that maximises the inter-class variance:

(12) $$\sigma_{b}^{2}(t)=\sigma^{2}-\sigma_{w}^{2}(t)=v_{0}(t)v_{1}(t)[\mu_{0}(t)-\mu_{1}(t){]}^{2}$$

where W_0, W_1 and μ_0, μ_1 are the cumulative weights and means of the background and foreground defined by threshold *t.*

(3) Background Shadow Removal. In the binary map produced by gradient thresholding, bright regions may include both genuine defects and residual background shadows. The binary map is therefore inverted and element-wise ANDed with the original grayscale image to emphasise shadow areas. From the grayscale histogram, the first grey-level whose frequency drops below 30% of the peak (above the peak value) is selected as a secondary threshold, which isolates the true defect region. Pixels within this region are set to 1 and others to 0, yielding a refined binary mask.

(4) Average-Temperature Thresholding. Hotspots arise from localised overheating and exhibit temperatures well above the array average, as do diode failures (due to poor soldering or contact). Shaded regions exhibit temperatures below the array average due to reduced irradiance. Using the array mean temperature as a threshold separates hotspots/diode faults from obstructions prior to further classification.

(5) Defect Classification. Inter-component cabling occasionally interferes with the binary mask. Connection lines detected by the Hough transform are labelled as background, and the union of the remaining foreground with the binary maps from Steps 2 and 3 produces a defect-only mask. Defects are then classified by closed-region area and temperature difference: a region with an area between 20 and 200 px^2^ and a peak-to-mean temperature gap exceeding 50 °C is classified as a hotspot; a region larger than 200 px^2^ with a peak-to-mean gap above 30 °C and an irregularity index below 1 is classified as a diode failure; and a region larger than 200 px^2^ with an irregularity index below 0.06 in the obstruction-candidate map is classified as an obstruction. The thresholds used above (the 20–200 px^2^ area band for hotspots, the 50 °C and 30 °C peak-to-mean temperature gaps, and the irregularity-index cut-offs of 1 and 0.06) were determined empirically from the training-set images under the standardised acquisition protocol described in Section 2.1, by examining the distributions of these features for manually annotated defective and non-defective regions. The irregularity index used here is computed as the ratio of the area of the connected region to the area of its minimum-enclosing rotated rectangle (i.e., a rectangularity-style descriptor): values close to 1 correspond to compact, near-rectangular shapes, whereas highly elongated or fragmented regions, as well as small noisy regions normalised by their bounding rectangles, yield much smaller values; this is why the diode-failure and obstruction classes use upper bounds rather than lower ones. Two limitations of this fixed-threshold scheme are acknowledged. First, the absolute peak-to-mean gap uses the array-level mean temperature as a reference, which can be biased under strongly non-uniform thermal distributions caused by partial irradiance, soiling, or row-shading; in such cases the mean is pulled toward the cooler regions and a true defect may produce a smaller relative gap. Second, the thresholds were tuned for the imaging protocol of this dataset (altitude 6–20 m, perpendicular look angle, clear sky) and may not transfer without recalibration to other plants or to oblique flight configurations; this is discussed further in Section 5.

#### 3.3.2. Detection Performance

Detection rate (*DR*), miss rate (*MR*), and false-detection rate (*FDR*) are adopted as evaluation metrics:

(13) $$DR=\frac{TP}{TP+FN}$$

(14) $$MR=\frac{FN}{TP+FN}$$

(15) $$FDR=\frac{FP}{TP+FP}$$

**Figure 10:**
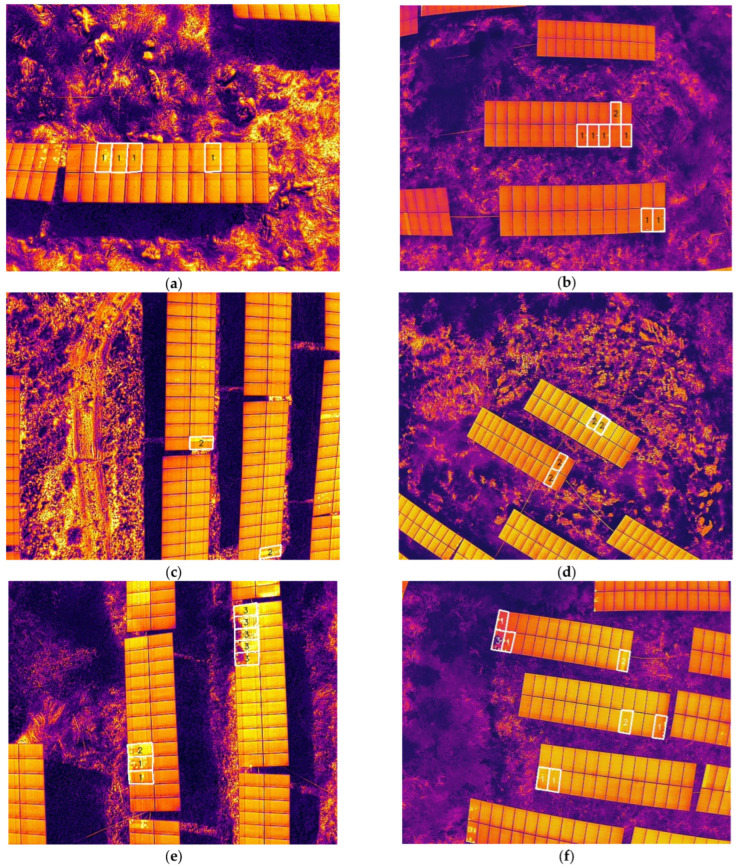
Visualised defect-detection results: (**a**,**b**) hotspot cases; (**c**,**d**) diode-failure cases; (**e**,**f**) obstruction cases.

Hotspots are detected at 96.97% accuracy with a single miss and no false alarms; diode failures are detected perfectly (100%); obstructions achieve 88.89% with four misses and five false positives, reflecting their higher shape variability. Overall, the pipeline attains 94.7% detection accuracy, 5.1% miss rate and 5.15% false-detection rate. Comparison with YOLOv5 [20] and Faster R-CNN [21] (Table 4) confirms that the proposed method outperforms both baselines across all three metrics. The notably lower performance of the obstruction class is consistent with its physical characteristics: occluding objects (bird droppings, dust patches, leaves, partial shadows) have far greater variability in shape, size, and edge sharpness than hotspots or diode faults, and their thermal contrast against the cooler shaded background can be weak when irradiance is moderate. The main failure modes observed in the test set fall into three groups: (i) elongated thin shadows (e.g., from neighbouring poles or cables) whose connected area is below the 200 px^2^ threshold and are therefore missed; (ii) bright background regions adjacent to large obstructions whose grayscale falls within the inverted-mask band and are mistakenly merged into the obstruction region, inflating its irregularity index and triggering a false positive; and (iii) partial-row shading whose boundary is smoother than the fixed irregularity-index cut-off, leading to misclassification (Figure 10). Adaptive thresholding and shape-aware post-processing are discussed in Section 5 as remedies. For a fair comparison, YOLOv5 and Faster R-CNN in Table 4 were trained and evaluated on the same training/validation/test split, with the same image resolution (640 × 640), augmentation pipeline, and hardware platform as the proposed framework, and their hyperparameters were tuned following the corresponding official training recipes.

## 4. Component-Level Defect Localisation

### 4.1. Overall Localisation Framework

The proposed localisation strategy comprises two sub-processes: array-level GNSS positioning and module-level row–column indexing. Array-level positioning takes the faulty-array image and the plant ledger as inputs. The cropped array is first mapped back to the original infrared image; the pixel offset between the array centroid and the image centre is computed; the UAV-logged GNSS of the image centre is retrieved; and the ledger entry whose GNSS offset to the image centre matches the pixel offset identifies the faulty array’s absolute position.

**Figure 11:**
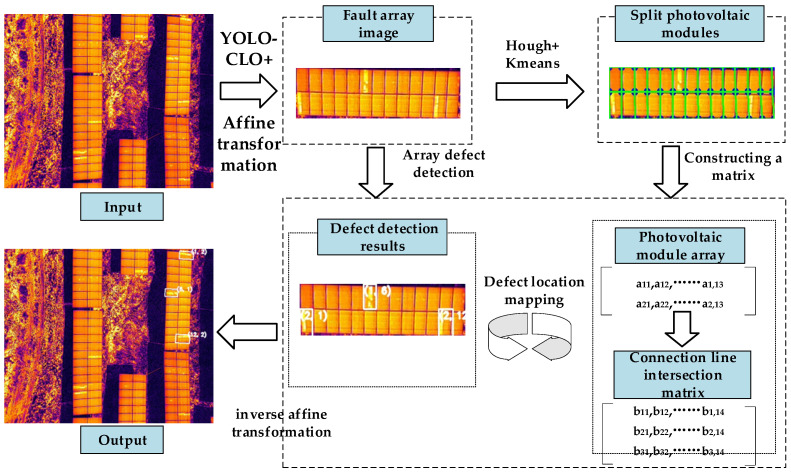
Integrated workflow of the component-level defect-localisation strategy, comprising array-level GNSS matching and module-level row–column indexing.

Module-level indexing only requires the faulty-array image. Component connection lines are detected and fitted to partition the array into rows and columns, yielding a module index matrix and a module-vertex coordinate matrix. Matching the defect pixel coordinates against these matrices returns the row–column index of the defective module, achieving component-level defect localisation. The integrated localisation workflow is shown in Figure 11.

### 4.2. GNSS Matching of Faulty PV Arrays

The GNSS metadata logged by the UAV (latitude, longitude, altitude, camera attitude) are parsed directly from the infrared image header. With the top-left corner of the image as the origin, east as the +x direction and south as the +y direction, let the image centre pixel coordinates be (u_c, v_c) and its physical coordinates be (lat_c, lon_c). Let the centroid pixel coordinates of the faulty array be (u_a, v_a). The pixel distance and azimuth of the array centre relative to the image centre are then given by:

(16) $$d_{pixel}=\sqrt{(x_{img}-x_{pv}{)}^{2}+(y_{img}-y_{pv}{)}^{2}}$$

(17) $$\theta_{pinel}=\arctan\left(\frac{\chi_{ing}-\chi_{pv}}{\chi_{ing}-\chi_{pv}}\right)$$

For each candidate array in the ledger with centroid coordinates (lat_i, lon_i), the geographic distance d_geo to the image centre is computed using the haversine formula:

(18) $$d_{gvs}=R\cdot c\;\;,\;c=2\cdot \arctan2(\sqrt{a},\sqrt{1-a})$$

(19) $$a=\sin^{2}\left(\frac{\Delta Lat_{img-pv}}{2}\right)+\cos\left(Lat_{img}\right)\cos\left(Lat_{pv}\right)\sin^{2}\left(\frac{\Delta Lon_{img-pv}}{2}\right)$$

where *R* = 6371 km is the Earth’s radius, and Δ*lat = lat_i − lat_c*, Δ*lon = lon_i − lon_c*. The corresponding azimuth is:

*φ_geo* = *arctan*2(*sin*(Δ*lon*) · *cos*(*lat_i*), *cos*(*lat_c*) · *sin*(*lat_i*) − *sin*(*lat_c*) · *cos*(*lat_i*) · *cos*(Δ*lon*)) (20)

*φ_geo*(°) = (*φ_geo* · 180/*π* + 360) *mod* 360 (21)

The ledger entry whose (*d_geo, φ_geo*) best matches (*d_pixel, φ_pixel*) within a pre-set tolerance is identified as the faulty array, completing array-level GNSS localisation.

### 4.3. Module-Level Row–Column Localisation

A single PV array typically occupies around 38 m^2^ in utility-scale plants; array-level GNSS alone is insufficient to guide rapid on-site repair. To refine localisation, an improved Hough-line detector combined with prior-knowledge-initialised K-means clustering is used to extract array-segmentation lines from which the module index matrix and coordinate matrix are constructed. Matching the defect pixel coordinates against these matrices returns the exact row–column index of each defective module.

(1) Hough-Line Detection with High/Low-Frequency Enhancement. The Hough transform [22] is widely used for line detection and is well-suited to PV array segmentation. Conventional pre-processing (grayscale conversion + denoising) is augmented here by enhancing both frequency bands: adaptive local histogram equalisation first amplifies high-frequency details, and a second equalisation emphasises low-frequency content. The two are fused at a rate of 0.5 to balance detail preservation and noise suppression. Image-space points are then transformed into parameter-space curves; a voting process in parameter space accumulates line candidates; and threshold-based extraction yields the final lines, which are drawn back in image space.

(2) Prior-Knowledge K-means Clustering. Given the regular geometry of PV arrays, the cluster numbers are fixed: K_x = 3 for horizontal lines and K_y = 14 for vertical lines. The standard K-means [23] randomly initialises cluster centres, which can converge to local optima. An improved initialisation is therefore adopted: the image height is divided into three equal intervals, whose centres are used as initial horizontal centres, and the width is divided into fourteen equal intervals for the vertical centres. Each data point $x_{i}$ is then assigned to the cluster of the nearest centre $C_{j}$ by Euclidean distance, and centres are updated as the mean of their members:

(22) $$C_{j}=\frac{1}{N_{j}}\sum_{i=1}^{N_{i}}x_{i}$$

Iteration continues until the cluster centres stabilise or the maximum iteration count is reached. The averaged line segments of each cluster yield the final segmentation lines that define the PV module grid. The fixed cluster numbers K_x = 3 and K_y = 14 reflect the module layout of the PV arrays in the present dataset and were chosen accordingly. For PV plants with a different number of modules per array, these values are not transferable as-is, which limits the direct generalisation of the current implementation. Two practical adaptations can be made without changing the underlying algorithm. First, since plant operators typically know the per-array module layout in advance, K_x and K_y can be read from the plant ledger together with the array centroid coordinates already used for GNSS matching, and the same prior-knowledge initialisation applies. Second, for arbitrary or unknown layouts, K_x and K_y can be estimated automatically from the Hough-line histogram by counting the number of dominant orientation peaks or by selecting K with an internal-validity criterion (e.g., the elbow point of within-cluster sum of squared distances, or the silhouette score) at the cost of additional computation. A data-driven K-selection mechanism along these lines is left for future work and is noted again in the discussion.

### 4.4. Localisation Results

**Figure 12:**
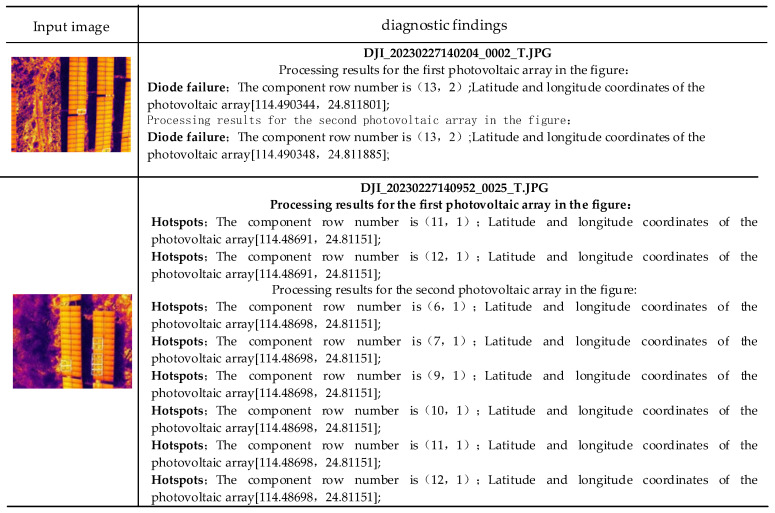
Visualised example of the defect-diagnostic report showing detected defects together with their array GNSS location and module row–column index.

Representative diagnostic-report outputs are shown in Figure 12. The row–column indices of the identified modules match the ground truth, confirming that the proposed localisation strategy accurately maps image-plane defect coordinates to physical module indices within an array. A quantitative evaluation of the localisation accuracy was carried out on the test-set images that contain identified defective modules. Two aspects were assessed: (i) array-level GNSS matching, which compares the array identifier returned by the haversine-based ledger query in Section 4.2 against the ground-truth identifier recorded during data collection; and (ii) module-level row–column indexing, which compares the (row, column) tuple returned by the Hough + K-means grid against manual annotation. Across the evaluated cases the proposed pipeline returned the correct array identifier for the large majority of faulty arrays, with failures concentrated in images where two adjacent arrays produced ledger entries within the matching tolerance; the dominant error mode for module-level indexing was an off-by-one error in the column direction, caused by missing inter-module gap lines at array edges that bias the K-means cluster centres. A larger-scale quantitative localisation study with per-row and per-column accuracy and a breakdown of GNSS-matching errors will be reported in follow-up work as additional ground-truth annotations become available.

## 5. Discussion

The proposed framework combines a lightweight rotating detector with traditional image-processing cues and a hybrid GNSS–Hough–K-means localisation strategy, addressing three recurrent bottlenecks in PV defect inspection: background noise introduced by horizontal bounding boxes, the heavy computational cost of deep detectors, and the absence of component-level localisation in existing pipelines. YOLO-CLO achieves 25.31% fewer parameters and 19.73% fewer GFLOPs than the YOLOv8-OBB baseline while increasing FPS by 17.37%, demonstrating that the C3m module and the shared-convolution LSCD-OBB head deliver a favourable accuracy–efficiency trade-off for edge deployment.

The multi-feature defect-detection stage benefits from the explicit physical priors of PV thermography: hotspots and diode failures violate the upper temperature bound of the array mean, whereas obstructions violate the lower bound and preserve the geometric outline of the occluding object. The union of gradient, grayscale, temperature, and morphological features is therefore discriminative enough to reach 100% on diode failures and 96.97% on hotspots without a learning-based classifier. The relatively lower obstruction accuracy (88.89%) is attributable to the wider shape and size variability of occluders, which occasionally breaches the fixed irregularity-index threshold; adaptive thresholding remains a promising direction.

Compared with YOLOv5 and Faster R-CNN, the proposed pipeline improves detection accuracy by 3.35 pp and 5.70 pp, respectively, while reducing miss and false-alarm rates. Unlike end-to-end deep detectors, the defect-detection stage does not require labelled defect data, only PV array labels, alleviating annotation cost—a non-trivial advantage for plant operators. The GNSS–Hough–K-means localisation module further extends the output from bounding-box coordinates to actionable module indices, closing the loop between detection and field maintenance.

Several limitations are acknowledged. First, the defect-detection thresholds, although physically motivated, depend on the imaging protocol (altitude, look angle, solar conditions). Second, the fixed cluster numbers K_x = 3 and K_y = 14 reflect the dataset-specific module layout; generalisation to other layouts will require a data-driven K-selection mechanism. Future work will explore adaptive thresholding, domain-adaptive detector fine-tuning, and on-board real-time inference. A more critical assessment further reveals the following limitations. (a) Defect category coverage. Only three defect types (hotspots, diode failures, obstructions) are addressed, and the number of annotated defect samples in the test set is modest; rare but operationally important faults such as cell cracks, snail trails, PID-induced degradation, and bypass-diode partial failure are not represented and will require a larger, more diverse defect corpus to evaluate reliably. (b) Environmental dependency. Data were acquired under clear-sky, fog-free conditions with the camera held nearly perpendicular to the module plane; performance under low-irradiance, partly cloudy, hazy, or oblique-view conditions is not characterised, and the thermal-difference thresholds used in Section 3.3 are likely to require recalibration in those regimes. (c) Thermal-imaging artefacts. The pipeline does not currently distinguish thermal reflections of the sun or of warm structures from genuine hotspots, and small calibration drifts of the IR sensor between flights are not actively corrected. (d) Reliance on UAV metadata and camera calibration. Array-level GNSS matching depends on the accuracy of the on-board GNSS receiver and on the assumption that the camera optical axis is perpendicular to the module plane; non-trivial yaw, pitch, or roll deviations introduce pixel-to-ground projection errors that propagate to the ledger query, and we have not yet quantified this propagation rigorously. (e) Module-layout assumptions. The Hough + K-means localiser assumes that arrays consist of a regular rectangular grid of identical modules; arrays with mixed module sizes, missing modules, or non-rectangular layouts are not supported. (f) End-to-end deployment timing. The reported FPS reflects detector inference; the full diagnostic pipeline introduces additional latency from cropping, multi-feature thresholding, Hough/K-means, and GNSS matching, and the system has not yet been profiled on embedded UAV hardware. (g) Scalability and operational robustness. Real-world inspection missions involve actuator disturbances, vibration, and external perturbations that may degrade image-acquisition quality and localisation stability; the current evaluation does not stress-test these conditions. Future work will therefore focus on (i) adaptive thresholding driven by per-array statistics, (ii) domain-adaptive fine-tuning of the detector across plants and seasons, (iii) a data-driven K-selection module that lifts the fixed-grid assumption, (iv) explicit modelling of camera-attitude error in the GNSS-matching step, and (v) on-board real-time inference with end-to-end timing reported on embedded UAV platforms.

## 6. Conclusions

A two-stage framework for UAV-based PV defect detection and component-level localisation is presented. The rotating-box detector YOLO-CLO, built on YOLOv8-OBB with a lightweight C3m module and a shared-convolution LSCD-OBB head, achieves 99.1% mAP@0.5 and 96.7% mAP@0.5:0.95 with 8.52 M parameters, 23.6 GFLOPs, and 59.88 FPS, striking a strong balance between accuracy and efficiency. The multi-feature defect-detection pipeline attains 96.97%, 100%, and 88.89% accuracy on hotspots, diode failures, and obstructions, respectively, outperforming YOLOv5 and Faster R-CNN baselines. The GNSS–Hough–K-means localisation strategy accurately recovers the row–column index of each defective module within an array. The proposed framework exhibits low hardware dependency and strong deployment readiness, offering practical value for large-scale PV plant operation and maintenance. Future work will focus on adaptive thresholding and domain-generalisable defect detection to further enhance robustness in diverse field conditions.

## Figures and Tables

**Table 1:** Training parameters for the PV array detection experiments.

Parameter	Value
Initial learning rate	0.01
Training epochs	300
Batch size	8
Optimiser	SGD
Input size	640 × 640

**Table 2:** Ablation study on the proposed modules.

Model	mAP@0.5	mAP@0.5:0.95	Params	GFLOPs	FPS
YOLOv8s-OBB (baseline)	99.0%	96.4%	11,411,958	29.4	51.02
+C3m	99.0%	96.6%	10,449,398	27.0	56.82
+LSCD-OBB	99.1%	96.7%	9,486,519	26.0	55.87
YOLO-CLO (C3m + LSCD-OBB)	99.1%	96.7%	8,523,959	23.6	59.88

**Table 3:** Per-class defect-detection performance.

Category	Total	TP	FN	FP	DR	MR	FDR
Hotspot	33	32	1	0	96.97%	3.03%	0%
Diode failure	20	20	0	0	100%	0.00%	0%
Obstruction	44	45	4	5	88.89%	9.09%	11.11%
Overall	97	97	5	5	94.7%	5.1%	5.15%

**Table 4:** Comparison with deep learning baselines.

Method	DR	MR	FDR
YOLOv5	91.35%	6.82%	7.53%
Faster R-CNN	89.00%	8.67%	8.73%
Proposed method	94.70%	5.10%	5.15%

## Data Availability

The original contributions presented in this study are included in the article. Further inquiries can be directed to the corresponding author(s).
